# A randomised controlled trial to assess the feasibility and acceptability of remote psychosocial and exercise interventions for people with lupus: The ADAPT feasibility trial

**DOI:** 10.1007/s00296-025-05959-4

**Published:** 2025-09-24

**Authors:** Melanie Sloan, Thomas A. Pollak, David D’Cruz, Wendy Diment, Michael Bosley, Elliott Lever, Farhana Mann, Benjamin Sloan, James Brimicombe, Stephen Morris, Felix Naughton

**Affiliations:** 1 Primary Care Unit, Department of Public Health and Primary Care, Strangeways Research Laboratory, University of Cambridge, Worts Causeway, Cambridge, CB1 8RN UK; 2 Faculty of Medicine and Health Sciences, University of East Anglia, Norwich, UK; 3 Institute of Psychiatry, Psychology and Neuroscience, King’s College London, London, UK; 4 Rheumatology Department, Guy’s and St Thomas’ Hospitals NHS Foundation Trust, London, UK; 5 Patient and Public Co-Investigators, London, UK; 6 Rheumatology Department, Whittington Health NHS Trust, London, UK; 7 Department of Psychiatry, University College London, London, UK; 8 Department of Mathematics, University of Leeds, Leeds, UK

**Keywords:** Systemic lupus erythematosus, Psychosocial, Exercise, Pilates, Feasibility, Trial

## Abstract

Limited psychosocial support is available for people with lupus despite the highly reduced quality of life. This study assessed the acceptability, feasibility, and effectiveness estimations, of three (two psychosocial, one exercise) interventions. Lupus patients (N = 124) were randomised to a control arm or one of three interventions delivered remotely over 8–12 weeks: (1) listening support (The Wren project), (2) online Pilates classes, and (3) a text message and video support programme. Online follow up surveys post-intervention and six-months post-baseline included validated instruments for depression (PHQ-8), fatigue (FACIT-F), resilience (CD-RISC), acceptability measures and our co-designed “ADAPT” measure. A subsample of participants completed qualitative interviews. Hedge’s g and linear regression were used to estimate effectiveness. All interventions were feasible in terms of recruitment, time, and costs, and met the pre-defined acceptability criteria of > 75% rating the intervention as acceptable/highly acceptable. Helpfulness ratings were highest for listening support with 89% rating it as often/always helpful (62% for Pilates and 52% for Text/videos). Proportions of participants reporting that the intervention had made them feel better mentally often/always was 71% for The Wren, 57% for Pilates and 48% for the text/video group. Qualitatively, the listening support participants valued the “*safe space”* to talk, and several of the exercise class participants reported improvements to physical and mental health. Although the text message and video programme was acceptable, feasible, and very low cost, 41% of participants would rather have received a different intervention. Suggested text/video adaptations included greater tailoring, particularly to stage of disease journey. Attendance was low for Pilates (only 55% attended > 50% of classes). Estimates of effectiveness favoured all interventions compared to control, although most improvements reduced with time. The interventions were feasible to deliver and acceptable to patients, with indications of potential effectiveness. Further studies are needed to determine effectiveness.

*Trial registration*: ISRCTN72406488.

**Supplementary Information:**

The online version contains supplementary material available at 10.1007/s00296-025-05959-4.

## Introduction

Lupus is associated with a high burden of physical and mental health symptoms[1–3]. Whilst mortality rates have drastically reduced in the past 75 years, and there have been recent advances in biologic pharmacotherapies to reduce disease activity [4, 5], people with lupus typically experience a high burden of mental health difficulties [1], debilitating fatigue [1, 2], and impaired quality of life (QoL) [1]. These can arise from the direct effects of the illness [6], from social consequences such as on work and relationships [7], and from the exigencies of their often prolonged and distressing diagnostic and treatment journeys [8].

Psychosocial support to improve QoL and assist people in adapting to life with lupus remains an essential, yet currently unmet, need. More support is urgently required, evidenced by recent research findings that almost 50% of lupus patients had experienced suicidal thoughts [9]. Psychosocial support can be the first casualty of increased physician time constraints [10], also exacerbated by the pressures on health systems of Covid-19. With Covid-19 now endemic, a degree of risk–and understandable caution regarding face-to-face contact among immunocompromised lupus patients [11]–is highly likely to continue long into the future, from both Covid and other infections. In addition to reduced infection risks, remote interventions may also be preferable to some patients where high levels of debilitating fatigue and mobility issues are prevalent, and to reduce geographical and socioeconomic access inequalities.

Two recent systematic reviews [12, 13] on holistic support for lupus patients demonstrated varied levels of effectiveness. Various types of counselling and psychotherapy, particularly CBT [13], have been trialled for autoimmune patients. Listening support without advice has not been trialled, and has been identified as an unmet need among patients informing this research. Listening support may assist people in adapting to their disease, and improve their mental health. Physical activity may improve both fatigue and mental health symptoms, and has been supported in a recent review that informs the 2021 European recommendations for lifestyle improvements in rheumatology patients [14]. Multiple small studies have demonstrated effectiveness of exercise in SLE, but recent reviews concluded that the evidence was weak, and the optimal–and safest–format unclear [15]. Another promising approach is digital communication tools, such as SMS text messaging, as a delivery mode for providing behavioural and psychosocial support to people with lupus remotely. Evidence indicates that digital support can be an effective and cost-effective way of helping people with long-term conditions to self-manage, including for type 2 diabetes [16] and asthma [17]. Digital support targeting QoL, behavioural and psychological outcomes has been under-investigated for lupus patients [12]. The text/video programme is anticipated to educate and provide psychosocial support, thus potentially improving knowledge, control and QoL.

As these different types of interventions are likely to address somewhat different aspects of SLE patients’ quality of life, combinations of interventions could be needed. However, before intervention combinations are explored, the impact and viability of such approaches needs to be determined. The aim of this study was to assess the acceptability, feasibility and potential effectiveness of three different remote interventions providing psychosocial or exercise support, to inform future effectiveness evaluations. The interventions selected included: (1) listening support (2) an online Pilates exercise class, and (3) a supportive and educational text message and video programme.

## Methods

### Study design

This was a four-armed randomised controlled feasibility trial comparing (1) listening support, (2) An online Pilates course and (3) a text messaging and video support programme to (4) a control group receiving usual care only. Randomisation followed a 1:1:1:1 allocation ratio with the listening support allocations having to cease at 30 participants due to capacity issues. An additional four participants had completed their expression of interest forms and were randomised to one of the remaining three groups. The statistician was blinded to allocation.

Intended sample size was N = 120 (30 per group), calculated on pragmatic and logistical grounds based on the capacity of two of the interventions (the listening support and the Pilates group). Due to capacity limitations of these intervention providers, the interventions were delivered over two approximately equal sized phases.

### Participants

Participants were recruited from the INSPIRE study survey [9], and online via lupus Facebook and forum groups. Inclusion criteria were being aged 18 or over, a UK resident, confirmation of a lupus diagnosis written on a clinic letter, having primary access to a mobile phone, being able (or willing to learn) to use Zoom, and physically able to do gentle Pilates exercise.

### Interventions

The three interventions were delivered remotely over 8–12 weeks:

### Listening support

The Wren Project is a charity which provides free online one-to-one emotional support to people living with an autoimmune disease. Their philosophy is not to counsel, rather to listen without judgement or advice. Participants were allocated to a volunteer who they were scheduled to speak to over video call for an hour every fortnight over 12 weeks. Volunteers had all completed The Wren’s intensive training programme in listening skills.

### Online Pilates class

The Flexifit Pilates group had two scheduled sessions per week over eight weeks, each lasting 50 min, via Zoom. Sessions were with the same trained instructor each time in groups of 15–16 and restricted to trial participants only. Sessions consisted of a warm-up, a balance section, a main exercise component which included strength exercises, stretch and cool down, followed by 5–10 min to informally chat as a group. Exercises were adapted for the needs of each individual.

### Text message and video support programme

The lupus-specific eight-week support programme consisted of between two and four texts per day including links to two videos per week (see Table 1 for programme structure and Table 2 for example texts). Participant’s names were included in approximately 25% of texts. The texts were written by the study team with external patient and clinician input, and the texts were signed from four main sources. Sources were named, introduced, and pictured in an intervention allocation letter (see supplementary information), and included a rheumatologist, behavioural scientist, fellow patient, and psychiatrist.

**Table 1:** Content and structure of the text messaging programme

Content	Structure
Overall structure	Aside from week 1 (introductions) and week 8 (weaning down support), the weeks were structured around Monday’s texts from FN initiating behavioural change (e.g. physical, social, medication, diet) and behaviour reinforcing/maintenance (goal setting and maintenance)
Focused texts	Two days each week had texts focusing on one of the major areas identified in the INSPIRE project^1^ as problems including: individual symptoms (e.g. cognitive, fatigue, anxiety), medical relationships, blood tests, and withdrawal from life. Some texts contained links to further resources, such as academic papers and mental health support charities
Personal experience	Peer support texts (WD) often re-iterated one of the team clinician’s messages from personal experience
Videos	Tuesdays were mental health video days from the team psychiatrist (TP). Participants also received an additional video per week from another provider

**Table 2:** Example texts from each provider

Provider and speciality	Example texts
**Psychology and behavioural science texts** Professor Felix Naughton, behavioural scientist	*Behavioural scientists use many techniques to help people make positive changes. They can seem basic, but they work. I'll share some in my upcoming texts.* Felix *Setting an 'action plan' can help with your tasks. A good action plan includes the What, Where, When and With whom. Breaking it down helps.* Felix
**Peer support** Wendy Diment, SLE patient and vice chair of LUPUS UK trustees	*Even on my bad days I try to get out with my dog. A 10-min gentle walk does help my mind, even if I feel extremely fatigued.* Wendy *After years of using various methods to remember my medications, I have now given in and bought a large pill box. Old before my time, but it works!* Wendy
**Psychiatry and mental health** Dr Tom Pollak, neuropsychiatrist	*Lupus can affect your mental health in many ways. It’s easy to say you’re OK when you’re not. Don't suffer in silence: if you are struggling, speak to your GP. Please click here for a video on lupus and mental health.* Dr Tom *Hi [name] Hope it’s one of the better days. On bad days sometimes it helps to just get from moment to moment. We’re here with you.* Dr Tom
**Rheumatology and disease specific advice** Professor David D’Cruz, lupus specialist	*Testing the urine is as important as blood tests for assessing lupus activity. Ask your doctor to test urine if they aren’t already doing this.* Prof D’C *Some people are on long-term steroids for their lupus. If you have been taking steroids for several months you must not stop them suddenly.* Prof D’C

### Control group

Control participants continued to receive their usual medical care, as with other groups, and did not receive any study related interventions.

### Procedure and measures

All surveys were completed online via Qualtrics. Baseline surveys were completed before randomisation. Follow-up surveys and subsequent reminders were sent via email or text link immediately following the end of the intervention or control period (eight weeks), and at six months post-baseline.

Survey content included sociodemographic and disease information (baseline), acceptability and intervention specific questions (post-intervention follow-up), and validated instruments and patient-designed questions (all time points).

Validated instruments were selected after consultation with patient groups as to which areas of life they felt the disease most adversely impacted and what they most hoped to improve in their lives. These included: depression (measured by the PHQ-8 [18]), fatigue (FACIT-F [19]), resilience (CD-RISC [20]) and loneliness (UCLA 6-item [21]), in addition to the EQ-5D-5L quality of life measure. Extensive pre-trial engagement with SARD patients identified that, while a range of validated measures of adaptation in chronic illness exist, none were considered appropriate for this patient population. We therefore co-designed with patients an “ADAPT” measure (Supplementary information, Figure A4), and trialled it within this study. ADAPT scoring was from 0 to 100 with 100 signifying the best level of adapting. The measure will be validated in future trials.

Feasibility was assessed through cost, recruitment, retention, intervention engagement and follow-up rates, and through discussions with the providers, participants, and research team as to the feasibility from their perspective. Progression criteria to a large-scale definitive trial were pre-defined in the protocol (https://osf.io/cjyb8).

Among intervention participants, acceptability was assessed through a one item Likert-type scale question on how acceptable they found the intervention, plus several additional measures of satisfaction/ dis-satisfaction (e.g. I would have preferred an alternative method of support).

Interviews (n = 13) were carried out by MS following the six month follow-up survey with purposively sampled participants to ensure a range of sociodemographics were represented from the three intervention groups to further explore acceptability and views on the interventions and procedures. Interviews were via Zoom and/or email correspondence.

### Analysis

The pre-specified protocol (https://osf.io/cjyb8) included a preliminary statistical analysis plan (SAP). Prior to accessing the data, the statistician (BS) produced a more detailed SAP (abbreviated details in supplementary information). Quantitative data were analysed using SPSS and R software.

Due to it being a feasibility study, confidence intervals as opposed to p values were calculated for the change in outcome measures for each intervention against the control group, with effect sizes based on complete case analysis calculated using Hedge’s g for validated instruments. Normality of the distribution of data was tested through the Shapiro–Wilk test. Linear regression was used for adjusted analyses, to demonstrate changes in outcome measures independent of the potential variance created by different demographics.

In addition to the quantitative measures, open ended survey responses and interviews with purposively sampled participants were analysed thematically to inform the process evaluation [32], and to further assess acceptability, feasibility and views on potential effectiveness.

#### Ethical approval

This manuscript reports phase 1 of a pre-registered trial (ISRCTN: 72406488). Ethical approval was obtained through the Cambridge University Psychology Research Ethics Committee (PRE.2023.026).

## Results

Participants were mostly female (97%), white (82%) and aged between 30 and 69 (91%). Almost half (47%) had been diagnosed > 10 years previously. In terms of job status, 31% were not currently working due to health (Table 3).

**Table 3:** Participant characteristics

Characteristic	Total (N = 124) (%)	Text/video group (n = 32) (%)	Listening support (n = 30) (%)	Pilates group (n = 31) (%)	Control group (n = 31) (%)
Age					
18- 29	3 (2%)	2 (6%)	1 (3%)	0 (0%)	0 (0%)
30–39	27 (22%)	5 (16%)	6 (20%)	9 (29%)	7 (23%)
40–49	29 (23%)	10 (31%)	7 (23%)	7 (23%)	5 (16%)
50–59	30 (24%)	7 (22%)	9 (30%)	8 (26%)	6 (19%)
60–69	27 (22%)	5 (16%)	5 (20%)	6 (19%)	11 (35%)
70 +	6 (5%)	3 (9%)	1 (3%)	1 (3%)	1 (3%)
Prefer not to say	2 (< 2%)	0 (0%)	1 (3%)	0 (0%)	1 (3%)
Gender					
Female	120 (97%)	30 (94%)	29 (97%)	31 (100%)	30 (97%)
Male	4 (3%)	2 (6%)	1 (3%)	0 (0%)	1 (3%)
Other	0 (0%)	0 (0%)	0 (0%)	0 (0%)	0 (0%)
Prefer not to say	0 (0%)	0 (0%)	0 (0%)	0 (0%)	0 (0%)
Ethnicity					
Asian	7 (6%)	2 (6%)	1 (3%)	3 (10%)	1 (3%)
Black	6 (5%)	3 (9%)	2 (7%)	1 (3%)	0 (0%)
White	102 (82%)	26 (81%)	22 (73%)	25 (81%)	29 (94%)
Mixed	4 (3%)	1 (3%)	2 (7%)	0 (0%)	1 (3%)
Other	2 (2%)	0 (0%)	1 (3%)	1 (3%)	0 (0%)
Prefer not to say	1 (< 1%)	0 (0%)	1 (3%)	0 (0%)	0 (0%)
Job status					
Working full time	35 (28%)	12 (38%)	11 (27%)	5 (16%)	7 (23%)
Working part time	25 (20%)	2 (6%)	8 (27%)	7 (23%)	8 (26%)
Student	2 (2%)	1 (3%)	0 (0%)	1 (3%)	0 (0%)
Retired	14 (11%)	5 (16%)	4 (13%)	3 (10%)	2 (6%)
Not currently working by choice	4 (3%)	0 (0%)	1 (3%)	2 (6%)	1 (3%)
Not currently due to health or took early retirement due to health	39 (31%)	11 (34%)	4 (13%)	11 (35%)	13 (42%)
Looking for work	3 (2%)	1 (3%)	1 (3%)	1 (3%)	0 (0%)
Prefer not to say	2 (2%)	0 (0%)	1 (3%)	1 (3%)	0 (0%)
Time since diagnosis					
< 1 year	8 (6%)	3 (9%)	3 (10%)	1 (3%)	1 (3%)
1–2 years	16 (13%)	5 (16%)	2 (7%)	4 (13%)	5 (16%)
3–5 years	19 (15%)	6 (19%)	5 (17%)	5 (16%)	3 (10%)
6–9 years	22 (18%)	6 (19%)	5 (17%)	4 (13%)	7 (22%)
10 years +	58 (47%)	12 (38%)	14 (47%)	17 (55%)	15 (48%)
Not sure	1 (< 1%)	0 (0%)	1 (3%)	0 (0%)	0 (0%)
Baseline validated instrument mean scores					
Depression (PHQ8)	11.45	11.90	10.73	11.25	11.90
Fatigue (FACIT-F)	32.50	33.77	30.03	33.47	32.61
Resilience (CD-RISC)	23.40	24.84	23.73	22.72	22.32
Loneliness (UCLA-6)	17.11	16.35	16.93	16.99	18.19
QoL (EQ-5d-5L)	12.69	13.13	12.07	12.66	12.87

### Feasibility

**Fig. 1:**
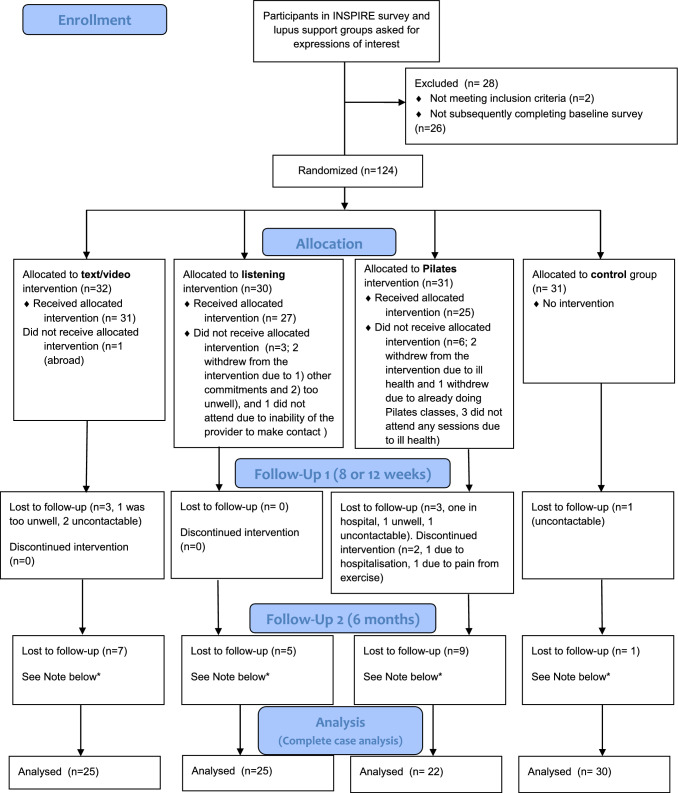
Consort flow diagram

The first group of 60 participants were recruited within three days, and the second group recruited within 30 days. Reasons for withdrawal, non-attendance and/or some non-completion of follow-up surveys were predominantly due to ill health. Follow-up rates for full or partial survey responses were 94% post-intervention and 86% six-months post-baseline (excluding pre-intervention withdrawals). (Fig. 1).

Attendance at The Wren was high with all participants (aside from two withdrawals prior to the intervention commencing, one DNA, and one who attended < 50% of the sessions) attending all or all but one session. Attendance was lower for Pilates with only one participant attending all 16 sessions and 55% of participants attending > 50% of the sessions. There were three withdrawals on allocation and three who did not attend any session. Interviews revealed that non-attendance was largely due to two reasons: 1) Several participants knew on sign-up that they were too physically unwell to participate in the exercise classes, yet still signed up with the hope that they would be randomised into one of the other interventions; 2) Other participants were physically well enough on sign-up, yet had disease flares or other illnesses/injuries during the eight week course that meant that they missed classes. In addition, many participants reported that the early class (9.30am) was too early for their morning joint stiffness and/or fatigue. The strong preference for afternoon classes was reflected in their higher attendance.

Engagement was difficult to measure for the text/video programme. Although there were no withdrawals in this arm, participants could have ignored the text messages and videos. However, 75% of participants followed up reported viewing all texts at least once.

### Intervention costs

Although The Wren provides their listening support for free to anyone in need and provided the intervention free of charge for this study, their costs include charity management and training volunteers. A contribution to the charity to fund extra staff and their training would need to be incorporated into a larger study. The cost to the charity per participant for 6 sessions is currently £102.

The Pilates instructor cost £800 per eight-week course for 15–16 people, giving a per person cost of £51.61.

The text message programme, via TextAnywhere, cost £10.25 per participant. The videos were free to upload through YouTube and the experts provided the videos free of charge.

### Acceptability

**Fig. 2:**
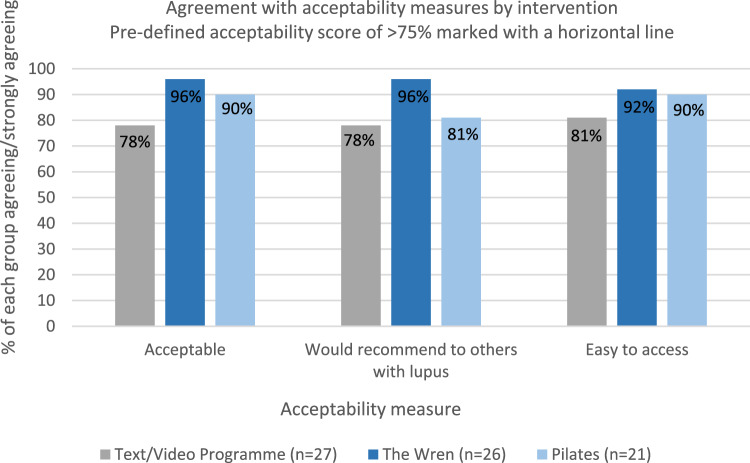
Acceptability measures by intervention

**Fig. 3:**
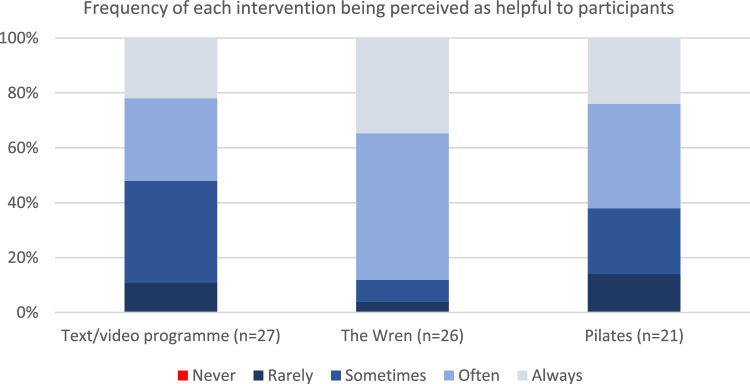
Perceptions of helpfulness of interventions. *Note*: These measures were optional after the validated tools and some attrition occurred due to length of survey

All interventions scored in the pre-defined “green” zone of > 75% for participant agreement that the intervention was acceptable (Fig. 2). All interventions also scored highly on ease of access (> 80% agreement), and > 50% of all participants considered that their intervention had been helpful “often” or “always” (Fig. 3). Helpfulness ratings were highest for listening support with 89% rating it as often/always helpful (62% for Pilates and 52% for Text/videos).

Participants more often reported that the interventions made them feel better mentally than worse (Supplementary figures A1/A2), with most stating they had never/rarely made them feel mentally worse. Proportions of participants reporting that the intervention had made them feel better mentally often/always was 71% for The Wren, 57% for Pilates and 48% for the text/video group.

Over half (52%) of the Pilates group stated that the classes often/always made them feel physically better (physical views were not requested for the other interventions as this was not an anticipated effect) with several detailing improvements in energy and pain. However, 15% of the Pilates group stated they sometimes felt physically worse from the class, and one participant reported that it had worsened her joint pain.

Most participants regarded the duration of the text/video programme and listening support as “about right” but the Pilates course as too short (Supplementary Figure A3). Several Pilates participants expressed a wish to continue post-trial and three joined the instructor’s usual classes, although other participants said that they could not afford the costs.

### Effectiveness estimates

Effect estimates for validated measures favoured the interventions over control at both time points. These were statistically significant, based on 95% confidence intervals, for all interventions for short-term depression and resilience improvements, compared to controls (Tables 4, 5, 6). There were also significant short-term reductions in fatigue for listening support and Pilates interventions, relative to controls. Most changes had reduced in effect size by the six-month follow up for most measures. Compared to controls, there were significant six-month improvements in resilience for both listening support and Pilates, and for quality of life for listening support. Results from the regression model are in supplementary information and are consistent with the unadjusted findings (Supplementary table A1).

**Table 4:** The Listening (The Wren Project) intervention compared to the control group

Outcome measure	The listening intervention	Control	Mean difference	95% CIs for difference	Hedge’s g
Depression: Baseline to F-up 1	− 2.96	0.30	− 3.26	**− 5.35, − 1.18**	− 0.816
Depression: Baseline to F-up 2	− 2.24	0.10	− 2.34	− 4.77, 0.09	− 0.513
Resilience: Baseline to F-up 1	1.43	− 1.20	2.63	**0.47, 4.79**	0.628
Resilience: Baseline to F-up 2	1.24	− 1.27	2.50	**0.06, 4.95**	0.544
Loneliness: Baseline to F-up 1	− 0.57	− 0.53	− 0.04	− 1.55, 1.47	− 0.013
Loneliness: Baseline to F-up 2	− 1.04	− 0.27	− 0.77	− 2.44, 0.89	− 0.251
QoL: Baseline to F-up 1	0.96	0.27	− 0.70	**0.36, 1.76**	− 0.308
QoL: Baseline to F-up 2	1.4	− 0.30	1.7	**0.35, 3.05**	− 0.689
Fatigue: Baseline to F-up 1	− 2.63	2.10	− 4.73	**− 8.60, − 0.86**	0.652
Fatigue: Baseline to F-up 2	− 2.12	− 0.17	− 1.95	− 6.56, 2.65	0.757

**Table 5:** Pilates intervention compared to the control group

Outcome measure	Pilates intervention	Control	Mean difference	95% CIs for difference	Hedge’s g
Depression: Baseline to F-up 1	− 3.12	0.30	− 3.42	**− 5.55, − 1.29**	− 0.867
Depression: Baseline to F-up 2	− 1.32	0.10	− 1.42	− 4.17, 1.33	− 0.292
Resilience: Baseline to F-up 1	2.72	− 1.20	3.92	**1.61, 6.23**	0.900
Resilience: Baseline to F-up 2	2.82	− 1.27	4.08	**1.31, 6.86**	0.828
Loneliness: Baseline to F-up 1	− 0.96	− 0.53	− 0.43	− 2.00, 1.15	− 0.146
Loneliness: Baseline to F-up 2	− 0.82	v0.27	− 0.55	− 2.35, 1.24	− 0.175
QoL: Baseline to F-up 1	− 0.04	0.27	0.31	− 0.79, 1.40	0.242
QoL: Baseline to F-up 2	− 0.77	− 0.30	0.47	− 0.93, 1.88	0.597
Fatigue: Baseline to F-up 1	− 2.56	2.10	− 4.66	**− 8.49, − 0.83**	− 0.666
Fatigue: Baseline to F-up 2	− 0.50	− 0.17	− 0.33	− 5.41, 4.74	− 0.039

**Table 6:** Text/video intervention compared to the control group

Outcome measure	Text/video intervention	Control	Mean difference	95% CIs for difference	Hedge’s g
Depression: Baseline to F-up 1	− 2.39	0.30	− 2.69	**− 5.20, − 0.18**	− 0.567
Depression: Baseline to F-up 2	− 2.12	0.10	− 2.22	− 4.90, 0.46	− 0.449
Resilience: Baseline to F-up 1	1.79	− 1.20	2.99	**0.58, 5.40**	0.639
Resilience: Baseline to F-up 2	0.60	− 1.27	1.87	− 0.71, 4.44	0.389
Loneliness: Baseline to F-up 1	− 1.79	− 0.53	− 1.25	− 2.84, 0.34	− 0.409
Loneliness: Baseline to F-up 2	− 0.56	− 0.27	− 0.29	− 1.87, 1.28	− 0.099
QoL: Baseline to F-up 1	0.74	0.27	− 0.47	− 1.64, 0.70	− 0.360
QoL: Baseline to F-up 2	− 0.44	− 0.30	0.14	− 1.28, 1.56	0.265
Fatigue: Baseline to F-up 1	− 0.26	2.10	− 2.36	− 6.73, 2.01	− 0.291
Fatigue: Baseline to F-up 2	0.32	− 0.17	0.49	− 3.84, 4.81	0.061

Depression (PHQ8), loneliness (RULS-6) and fatigue (FACIT-F) scores decreasing signifies improvement in these domains. Resilience (CD-RISC) and QoL (EQ5D-5L), scores increasing signifies improvements

Tables 4, 5, 6 Comparison of mean within person changes (complete case analysis) between each intervention group and the control group from Baseline to each follow-up for validated instruments.

### The ADAPT instrument

As with the validated instruments, many of the significant improvements in the ADAPT domains identified immediately post intervention at follow-up 1 were not sustained at the same level by follow-up 2 (Tables 7, 8). The highest within-person improvements from Baseline to Follow-up 2 were seen in the domains of “knowledge” for the text/video participants and “coping mentally” for the listening support participants.

**Table 7:** Changes in ADAPT instrument scores (+ Confidence intervals for mean difference in change between each intervention and control) between Baseline and Follow-up 1

ADAPT item	Text intervention	Listening support	Pilates	Control
Adapted to disease	0.04 (− 11.40, 11.74)	6.82 (− 4.68, 18.59)	9.72 (− 2.22, 21.92)	− 0.13
Feeling in control of life	**13.19 (4.16, 30.27)**	5.14 (− 2.63,20.98)	**14.24 (5.92, 30.63)**	− 0.40
Confidence to self-manage	4.33 (− 1.66, 15.93)	**9.25 (3.11, 20.99)**	**7.12 (0.12,19.72)**	− 2.80
Coping mentally	1.41 (− 8.37,15.45)	**11.21 (1.86, 24.83)**	**10.96 (1.81,24.38)**	− 2.13
Self-esteem	6.59 (− 2.76, 16.68)	3.36 (− 6.71, − 6.70)	**11.20 (0.94, 22.19)**	− 0.37
Knowledge of the disease	7.44 (− 3.29, 16.18)	2.57 (− 6.06, 9.20)	9.04 (− 1.38, 15.46)	1.00
Satisfaction with life	**10.30 (0.50, 19.90)**	8.25 (− 0.53, 16.83)	**11.84 (1.85,21.63)**	0.10
Participation in life	7.74 (− 1.41, 17.29)	8.54 (− 0.62, 18.09)	**10.08 (0.50, 20.06)**	− 0.20
Feeling part of a supportive community	9.59 (− 6.12,19.50)	4.00 (− 11.55, 13.75)	**12.60 (2.18,21.58)**	2.90

**Table 8:** Changes in ADAPT instrument scores (+ Confidence intervals for mean difference in change between each intervention and control) between Baseline and Follow-up 2

ADAPT item	Text intervention	Listening support	Pilates	Control
Adapted to disease	5.52 (− 7.30,14.48)	6.56 (− 4.81, 14.07)	5.86 (− 8.28,16.14)	1.93
Feeling in control of life	10.28 (− 4.12,19.95)	8.44 (− 4.96, 17.11)	6.05 (− 9.57, 16.93)	2.37
Confidence to self− manage	5.00 (− 7.37,16.50)	9.08 (− 0.37, 17.66)	6.18 (− 4.86, 16.36)	0.43
Coping mentally	5.24 (− 7.17, 15.65)	11.76 (− 0.74, 22.26)	3.91 (− 10.53,16.35)	1.00
Self-esteem	9.72 (− 3.13, 20.90)	1.12 (− 11.11, 11.68)	3.73 (− 8.82,14.61)	0.83
Knowledge of the disease	13.20 (− 0.02,19.35)	2.64 (− 8.03, 6.25)	5.68 (− 7.62, 11.92)	3.53
Satisfaction with life	9.64 (− 5.07, 18.15)	6.20 (− 8.06, 14.26)	3.68 (− 12.48,13.65)	3.10
Participation in life	4.60 (− 8.60, 18.46)	5.12 (− 5.10, 16.01)	3.36 (− 8.26, 15.65)	− 0.33
Feeling a part of a supportive community	6.72 (− 11.19, 19.36)	8.28 (− 10.98, 22.27)	9.32 (− 9.15, 22.52)	2.63

Each ADAPT item is scored from 0 to 100, and was measured by self-report at Baseline and Follow ups

Tables 7, 8 Mean within person changes for each group for the ADAPT domains.

### Process evaluation and qualitative analysis

#### Implementation: fidelity, dose, and reach of each intervention

The listening support sessions were provided by several different volunteers working for The Wren. Therefore, there would have been variations in delivery although these were likely to be minimal due to consistency of training and strong ethos. With one Pilates instructor only, the exercise intervention was delivered consistently. All sessions were provided as planned, although reach and dose varied due to many participants having fluctuating disease and attendance levels. The text/video intervention was automated so delivered consistently to all participants, although two participants reported that texts had not been delivered whilst they had been on holiday abroad.

### Effectiveness and potential mechanisms of effect

The potential mechanisms of effect for each intervention were explored in interviews and open-ended survey responses. These elicited very few negative comments regarding the listening support, other than a minority of participants expressing a preference for a more directive or suggestion-based approach. Listening support participants were largely focused on the improvements to their mental health and a high level of gratitude was consistently expressed for the provision of a *“safe space”* to be listened to without judgement or without feeling they were burdening family/friends or had to reciprocate:

“This is the only space I've ever had to talk about lupus in a totally open and honest way without feeling like I'm taking up someone's time… it was so refreshing and needed” (Ppt 002, 30's).

Several Pilates participants reported indications of positive effect in multiple domains aside from physical health including mental health and mood, resilience, and community, as explained by one participant:

“It was the first time I had the opportunity to engage with others with lupus which made me feel less alone. My body and mind feel stronger, and I have a higher sense of self-worth for taking part…The whole experience has made me feel happier (Ppt 001, 40's).

Although 41% of people receiving the text/video programme stated that they would rather have received a different intervention, there were indications of potential effectiveness raised in interviews. Some reported that the increased knowledge then led to empowerment and increased coping and feeling of control and increased confidence in communication with their clinicians:

“Explanations given in the texts and videos made it easier to cope with the disease because I had a better understanding of it” (Ppt 016, 40’s).

However, acceptability was reduced by participant irritation with some reports that some texts had a “*patronising*” tone leading to disengagement, and a common view among patients with longer disease duration that some of the advice was “*a little teaching gran to suck eggs”* (Ppt 34, 40's). Many participants had already attempted to modify their lifestyle to help manage their disease, and often reported finding the behaviour change texts unhelpful or irritating.

One particular criticism of the text programme was in encouraging participants to seek good medical care, including contacting their clinicians and obtaining a second opinion. Participant feedback was that this was “*out of touch”* with the reality of the current NHS situation, and an unobtainable ideal for most:

“I found the texts encouraging you to share with your doctors or tell them things difficult and sometimes annoying as it is hard on the outside world to contact a lot of those people” (Ppt 020, 30's).

### The importance of the intervention providers’ personality

An important finding was the key importance of the providers’ personality in influencing acceptability and potentially influencing effectiveness. This included empathy, approachability, and demonstrations of care and concern for the participants.

Without exception, extremely positive terms were used to describe The Wren listening volunteers and the feelings engendered (e.g. “*lovely”, “trust”, “safe”, “non-judgmental*”) with participants expressing views that the support had positive impacts:

“I hold in my heart the kindness I was shown by the Wren people, and I know how much my feelings are improved by having someone who understands lupus and believes in me” (Ppt 56, 60's).

Feedback from those attending the Pilates classes was also very positive, and usually incorporated positive comments about the exercise instructor’s personality “*Fantastic instructor, really empathetic & motivating”* (Ppt 040, 50’s) and “*The online Pilates instructor was absolutely lovely and very good at what she does. She made us all feel very welcome”* (Ppt 013, 40's)*.* Multiple participants used the terms “*lovely*”, “*approachable*”, “*bubbly”* and “*kind*” when referring to the instructor.

The text/video interventions also often engendered a sense of being cared about despite being widely recognized as generic: *“felt as if someone gave a damn. Thank you, it made a difference to me”* (Ppt 109, 60's*).* This was reported to be increased by each specialist being named on texts, visible on videos, and detailed in the introductory material with a biography and photograph. The video element was received very positively and participants appreciated feeling a connection with the providers, for example:

“Loved Dr Tom’s videos, he was so friendly and it felt like I was talking to someone…upbeat and supportive” (Ppt 081, 50's).

### Outcome measures for future effectiveness trial

Patients ordered the following in terms of their top 3 priorities for are of improvement: 1) Fatigue (72%), 2) Depression (61%), 3) Cognitive dysfunction (51%), 4) Adapting to having a chronic disease (44%), 5) Loneliness (41%) and 6) Anxiety (35%). Most participants (57%) ranked reducing fatigue as their number 1 priority.

## Discussion

This study found high levels of feasibility and acceptability for three remotely delivered interventions targeting psychosocial and quality of life outcomes. Feasibility of study recruitment and engagement was demonstrated by rapid recruiting times and high follow-up rates. Furthermore, scalability evidence for a definitive trial and implementation as an adjunct to usual care included ease of access for participants, low cost, and ease of delivery for the research team and intervention providers. While all three interventions met the pre-specified progression criteria for acceptability, findings indicated several areas of improvement for the text message and video support programme including greater tailoring, particularly to stage of disease journey. What was considered acceptable and helpful to a newly diagnosed participant was often viewed as patronising to the more experienced patients. Estimates of effectiveness favoured all interventions compared to control, although most improvements reduced with time, aside from resilience for Pilates, and both resilience and QoL for listening support.

This is the first trial to evaluate the type of listening support provided by The Wren charity. The Wren is the intervention that is least modifiable given it is an established service focused primarily on listening as opposed to counselling. It also had the highest levels of acceptability among all interventions tested. It was clear that more stringent physical health exclusion criteria would be required for the definitive trial for the exercise intervention, and a potentially higher level of withdrawals and non-adherence compared with the other interventions must be anticipated in any sample size calculations. This is due to the relapsing–remitting nature of lupus [7] and other autoimmune diseases, the timings of which are unpredictable. Findings indicated that in a future definitive trial, separating the exercise intervention from psychosocial interventions may be preferable by increasing the likelihood that only those who are physically able to participate sign up, thus increasing attendance. An alternative may be to have exercise class options that allow for varying levels of mobility, such as Tai Chi [22], or chair based Pilates or yoga classes [23]. Any form of exercise will still be contra-indicted for some patients at certain times. Short duration (8–12 weeks) exercise classes may encourage the development of longer-term exercise habits and skills to engender more confidence with self-initiated physical activity, or to subsequently join other activities to improve physical and mental health. However, a recent review found that increases in physical activity from interventions were often not sustained [24]. Future trials should therefore include approaches to promote the maintenance of physical activity, and account for any inequalities relating to accessing classes post-intervention, such as limited personal finances.

Despite the mechanisms of effect remaining uncertain, exercise has been identified as a “potential therapeutic tool in counteracting systemic inflammation” [25], which can contribute to fatigue and mental-health problems. Improvements in debilitating fatigue [1, 9] was the number one patient priority, and there were indications from the qualitative findings that some Pilates patients felt their fatigue had improved, supported by the post-intervention, although not six-month, follow up findings, relative to controls. However, our study was not designed to assess effectiveness and so would have had low statistical power to detect anything but large effect sizes. Depression is also prevalent in this patient group, and reduced in the Pilates group, in line with findings from a recent systemic review of exercise [26]. Our study’s group-based exercise class was found to incorporate social benefits in addition to physical ones, which may be particularly valuable to patients given their restricted participation in daily life [27]. Future studies are required to understand how much of the effectiveness is about reciprocal connections with other human beings and how much is specific to the content of the interventions. This study builds on recent publications on the importance of physical activity and tailoring [28], and on using technology for self-management in rheumatic diseases [29].

While in some cases face-to-face interventions may be superior/preferred to remote interventions, a review of rheumatology interventions found home-based physical activity had an equal effect to in-person interventions [30]. Our patient partners and wider patient consultations identified a preference for remote support, primarily for health and accessibility reasons. Our findings are similar to a very small prior study of remote supervised exercise classes where participants found them acceptable with small improvements in fatigue [31]. Our exercise class and Wren participants developed a close bond with their providers that did not seem to have been constrained by only meeting remotely over Zoom. A concern with the text/video programme was that, with the increasing use of Artificial Intelligence including highly interactive Chatbots [32], texts may be seen as old-fashioned and obsolete. However, our participants felt that they knew, and were supported by, the person behind the texts due to the method of using pre-introduced and photographed “sources” and largely appreciated this connection.

Selecting one primary outcome measurement for a future definitive trial is challenging due to the different anticipated and observed effects of each intervention. The differences in improvements in the different validated instruments and the co-designed ADAPT instrument domains between interventions suggest that combining these interventions into one complex intervention may be the most effective approach and provide holistic support. Whether the improvements engendered by each intervention in each domain will be additive if combined requires investigation. It is likely that more intensive support, such as an educational, supportive “adapting” course, is required in addition to the interventions trialled, particularly for those with the most distress and those newly diagnosed. Outside of the trial context, empowering patients to select the interventions and activities of most relevance, acceptability and interest to them would be the ideal.

### Strengths and limitations

A strength of this study team is the level of engagement and immersion in the long-term condition communities, with patient partners equal members of our research teams, including as co-authors of this article. The multidisciplinary team ensured variation and depth of knowledge and experience in designing and evaluating these interventions. A limitation was that we will not have achieved a wholly representative group due to online recruiting attracting certain sociodemographic groups and excluding others, particularly the most disadvantaged [33]. The gender distribution was highly skewed to female participation (97%), thus reducing the generalisability to other genders. An additional limitation was that the exercise classes were run only during the daytime. This may have excluded those working full time and thus may have skewed the results as the participants available to attend during the day may differ in multiple characteristics (e.g. disease severity) from those who were unavailable. Due to capacity limitations restricting the number of listening support participants to 30, randomisation of the final four participants was restricted to the remaining three groups. Duration of the interventions also varied between 8 and 12 weeks, although we were not directly comparing the interventions with each other. Although we attempted to ascertain reasons for non-participation, and losses to follow-up, this was not always possible due to non-response.

In addition, although acceptability rates for the Pilates course were high, these must be viewed with the caveat that those who did not attend many sessions also had higher rates of non-completion of follow-up surveys, and may have given lower acceptability scores. The highly positive effect of the exercise provider herself must be considered in terms of scalability, and it will be essential to trial multiple exercise providers to assess/mitigate the impact of different personalities and inter-personal skills. Continuity and sustainability of positive effects beyond the trial period was not planned for in this feasibility study, but have been considered when designing the full ADAPT trial and in implementation. This includes an additional 8 weeks of free classes and later signposting to continuation of classes. Although the co-produced ADAPT tool demonstrated indications of effectiveness in this population, its validity and reliability were not assessed in this study. The ADAPT instrument’s validity will be assessed as part of phase two of the ADAPT study, with further measures of effectiveness.

A limitation from having multiple diverse interventions for randomisation was that several participants signed up for the trial aware that they were too unwell for the exercise intervention but hoping to be randomised to a non-exercise intervention. This reduced participation in the exercise intervention and increased the likelihood of potential bias in the results.

## Conclusion

The three remote psychosocial or exercise interventions achieved the pre-determined acceptability and feasibility criteria for progression to a definitive trial, with indications of effectiveness favouring the interventions over the control group. The biggest barrier to successful interventions in this patient group is the often severe and fluctuating level of disease activity. This can render attendance at activities, particularly involving exercise, impossible at times. The Wren listening support and the Pilates classes can proceed unaltered aside from more stringent exclusion criteria for the exercise component and minor logistical adaptations. The text/video programme would benefit from alterations, to be agreed with patient groups, particularly greater tailoring to the length of time since diagnosis. However, each individual intervention is unlikely to have a positive effect on every one of the diverse areas requiring support for people with lupus and other long-term conditions. A complex combined multi-stage intervention is being developed by the authors to offer holistic support for lupus patients.

## Supplementary Information

Below is the link to the electronic supplementary material.

Supplementary file1 (DOCX 274 KB)

## Data Availability

Anonymised data will be available on reasonable request for research purposes.
