# Supplementary material for: A randomised controlled trial to assess the feasibility and acceptability of remote psychosocial and exercise interventions for people with lupus: The ADAPT feasibility trial
Source: Rheumatol Int. 2025 Sep 24;45(10):233. doi: 10.1007/s00296-025-05959-4 (PMC12460490; doi:10.1007/s00296-025-05959-4)
Supplement: Supplementary file 1 — Supplementary file1 (DOCX 274 KB) [file 296_2025_5959_MOESM1_ESM.docx]

**Supplementary information for: A randomised controlled trial to assess the feasibility and acceptability of remote psychosocial and exercise interventions for people with lupus: The ADAPT feasibility trial**

**Contents:**

Statistical analysis plan – pages 2-6

Intervention allocation letter for the text/video intervention group – pages 7-8

Figure A1. Frequency of each intervention making participants feel better mentally – page 9

Figure A2. Frequency of each intervention making participants feel worse mentally – page 9

Figure A3. Views on intervention duration – page 9

Table A1. Regression analysis:changes in validated instruments after controlling for demographics, page 10

Figure A4 – The ADAPT survey instrument – page 11

Supplementary references – page 12

**Statistical analysis plan**

# Aim

The aim is to assess the acceptability, feasibility and effectiveness of methods of remote psychosocial support for SARDs patients.

# Objectives

1. To trial multiple quality of life and mental health measures to determine the most appropriate primary and secondary outcomes for phase 2.
2. To test processes to inform phase 2 effectiveness trial.
3. To develop and trial our own ADAPT instrument to more accurately reflect, measure and monitor SARD patient personal, social and medical satisfaction.
4. To assess and estimate (phase 1) and compare (phase 2) the effectiveness of remote psychosocial interventions on the mental health, wellbeing, self-esteem, resilience, loneliness and disease acceptance of patients with systemic autoimmune rheumatic diseases.

# Participants

Participants were recruited from those expressing an interest on the related INSPIRE study survey^1^, and online via SLE Facebook and forum groups. Inclusion criteria included being aged 18 or over, being resident in the UK, confirming that they have an SLE diagnosis written on a clinic letter, having a mobile phone that they could receive text messages on, being able (or willing to learn) to use Zoom, and physically able to do gentle Pilates exercise.

Study design and size

A four-armed randomised controlled feasibility trial comparing 1) The Wren Project listening support, 2) An online exercise course and 3) a text messaging support programme to 4) a control group receiving no intervention. Randomisation to follow a 1:1:1:1 allocation ratio with The Wren allocations ceasing at 30 participants due to capacity issues. The Cambridge University Department of Public Health senior data manager (JB) will carry out the randomisation. Due to the nature of the interventions, it is not possible for the participants, the intervention providers, or the researchers carrying out interviews to be blinded to allocation. The statisticians (BS and SN) will be blinded to allocation. Participants who were not available at the pre-decided (daytime) Pilates class times were not included in the randomisation to the Pilates group.

Intended sample size is n=120 (30 per group), calculated on pragmatic and logistical grounds based on the maximum current capacity of two of the interventions (The Wren and the Pilates group). Due to capacity limitations of these intervention providers, the interventions will be delivered over two approximately equal sized phases.

# Outcomes

## **Acceptability Outcomes**

The primary acceptability outcome is a one-item question on whether the participants found the intervention acceptable, with the 5 options of: strongly disagree, disagree, neither agree nor disagree, agree and strongly agree. The percentage stating ‘agree and strongly agree’ will be combined and reported descriptively for the acceptability measure.

Additional measures of acceptability were measured with the same 5 item options, and the results will be reported descriptively as above and separately. These include the level of agreement with the following about the intervention: 1) helpfulness, 2) ease of access, and 3) would recommend to others with lupus.

Acceptability will also be assessed via the level of engagement with the interventions, proportion completing Follow-ups, and the proportion withdrawing. Engagement with The Wren and Pilates interventions will be assessed via the percentage of sessions attended (with the caveat that in this population with relapsing-remitting symptoms, non-attendance may be due to being unwell and not indicate lack of acceptability – this will be explored via interviews. Assessing engagement for the Text intervention will be via survey questions on how many texts and videos the participant read/watched.

A measure of unacceptability included: 1) would have preferred a different intervention.

Acceptability will also be assessed qualitatively via interviews and open-ended questions on the follow-up surveys

**Potential Effectiveness Outcomes**

1. The UCLA 6 item Loneliness scale (RULS-6^2^) – Adapted from the RULS loneliness scale which included 20 items with the questions that appeared most important. Participants are required to answer the new modified 6 items with the options of: Never, Rarely, Sometimes and Always. The higher the score, the greater the loneliness.
2. The Connor Davidson resilience scale (CD-RISC – 10 item)^3^ – There are 10 items in this self-rated assessment of stress coping ability. It was developed based on various concepts including; hardiness, adaptation, stress endurance. The higher the total score, the higher the resilience.
3. PHQ-8^4^ – An eight item depression scale. Each item consists of a scale that ranges from 0 to 3, with the PHQ-8 score being the sum of the items, leading to an overall scale of 0 to 24. A high score indicates higher severity of depressive symptoms.
4. FACIT-F^5^ – Designed to measure fatigue, this 13 item scale uses a 4 point Likert-scale and is split into two main parts: the impact of the fatigue on various aspect of life and the severity of the fatigue. A higher score indicates more severe fatigue
5. EQ-5D-5L^6^ – A self-rated assessment that measures the participant’s health related quality of life. Participants are questioned across 5 items with each item consisting of 5 response levels: no problems, slight problems, moderate problems, severe problems, and extreme problems.
6. ADAPT Instrument – Our own patient-designed instrument with various measures that may indicate the degree to which patients with chronic disease have adapted to – and reached a level of satisfaction with – their lives. There are 3 sections to this instrument comprising: Satisfaction with life (questions include: coping mentally, participation in everyday life), satisfaction with social support, and satisfaction with medical care. The ADAPT instrument is being trialled, and requires future validation, and will be able to be analysed and presented as individual section scores. Satisfaction with medical support was not considered to be influenced by the interventions in the ADAPT study so will not be included.

Participants will also be asked the degree (5 options from never to always) to which their intervention made them feel: 1) Better mentally, 2) Better physically, 3) Worse mentally, 4) Worse physically.

## **Feasibility Outcomes**

Feasibility will be assessed both qualitatively (with participants and providers) and quantitatively (from participant surveys, and data on recruitment speed and rates, and follow-up rates), and include assessments of: demand for the interventions, implementation, practicality, ease of adapting/ modifying for different populations/groups, integratability into usual care and expandability, and early effectiveness measures and what constitutes meaningful change for participants.

# Progression criteria

Progression to a full trial will be based on the following criteria. This decision will be made separately for each arm.

1. Green: >75% participants are satisfied/very satisfied with the support and engaged with the support.
2. Amber: 50-75% of participants are satisfied and engaged with the support.
3. Red: <50% of participants are satisfied and engaged, or where >25% of participants find the intervention unacceptable

Satisfaction level is measured using the one-item acceptability question, with consideration given to the secondary measures of acceptability and unacceptability, and responses from interview participants regarding areas of interventions that they found particularly acceptable and/or unacceptable.

Engagement level is measured as the number of participants who completed >50% of the intervention sessions, and the proportion completing both follow-up surveys of the number of participants signed onto the intervention groups.

# Sample Size

The total sample size is N=120 with n=30 in each of the 3 intervention groups of text messaging intervention, listening support (The Wren Project) and the online physical activity course (Flexifit Pilates) and n=30 in control

# Analysis Plan

## **Objective 1**: To trial multiple quality of life and mental health measures to determine the most appropriate primary and secondary outcomes for phase 2

Indications of potential effectiveness of each of the 6 listed validated instruments will be assessed by comparing the within-person change between Baseline and Follow-up 2 (6 months post baseline), with changes from Baseline- follow-up 1 also reported. Intervention changes will be compared to the control group using confidence intervals. Hedge’s g and linear regression will be used to estimate differences in effectiveness measures.

Appropriateness will also be determined qualitatively by eliciting participants’ views as to content and relevance of each outcome assessment, and their preferences elicited.

**Objective 2:** To test processes to inform phase 2 effectiveness trial. Process assessment will include: recruiting ease (measured by time taken to recruit sufficient numbers for the trial and amount of time and researcher involvement required), consistency of delivery, provider and recipient views of the processes and procedures as to acceptability and any difficulties requiring consideration for changing for phase 2.

## **Objective 3:** To develop and trial our own ADAPT instrument to more accurately reflect, measure and monitor SARD patient personal, social and medical satisfaction

## At phase 1, this will consist of a qualitative assessment of participants views of the ADAPT tool, suggestions for improvements and the % completing the ADAPT tool survey within the follow-up surveys.

## **Objective 4:** To assess and estimate (phase 1) and compare (phase 2) the effectiveness of remote psychosocial interventions on the mental health, wellbeing, self-esteem, resilience, loneliness and disease acceptance of patients with systemic autoimmune rheumatic diseases.

The effectiveness outcomes at 6 month post baseline will employ a multiple linear regression model fitted to 6 months post baseline, with covariates entered for age, time since diagnosis and intervention type. The difference in mean, and corresponding 95% confidence intervals will be reported. Complete case analysis will be used.

# Interim Analyses

No formal interim analysis will be undertaken. Descriptive analyses of recruitment, retention and other feasibility outcomes collected by the study team at various points during and after completion of the trial.

# Exploratory analysis

Depending on the socio-demographic composition of participants, further comparisons may be relevant (e.g. comparison of views and effectiveness of interventions by gender and/or age group and/or time since diagnosis)

# Practical steps to minimise bias

Statistical analysis will be conducted in line with this pre-defined plan to minimise the risk of data-driven bias.

Due to the nature of the interventions participants cannot be blinded. The statisticians will however be blinded.

**Intervention allocation letter for the text/video intervention group**

**The ADAPT research study**

Thank you very much for signing up to this research study where we are testing different methods of support to see if we can improve the lives of people with lupus.

**Allocated group – The Text/video message programme**

You have been randomly allocated to receive the text message programme.

**What will happen**

You will receive regular text messages/videos over 8 weeks from 4 different experts (see next page for their details). Texts will start in early September, and you will receive approximately three messages a day. There will also be links to videos from these experts and their colleagues.

**What you need to do please**

We will send you another survey in 8-12 weeks to see how you are and what you thought about the texts, and another survey 3 months later. We will also ask about your quality of life and health.

**What if you don’t want to have the texts or want to stop them part way through?**

You can withdraw at any stage without giving a reason. Your doctors do not know you are participating in this study, and it won’t affect your medical care at all whether you participate or withdraw at any stage. You can stop the texts at any time by texting STOP to the number provided. If you withdraw from the text messaging programme, we will keep any data we have collected to that point and will send you the follow-up surveys. This is because it’s important to get everyone’s views, whether they are good or bad. However, you do not have to complete the follow-up surveys if you don’t want to or if you choose to withdraw from the whole study.

**Any further questions?**

If you have any questions now or at any stage in the study, please contact the lead researcher, Melanie Sloan on [mas229@medschl.cam.ac.uk](mailto:mas229@medschl.cam.ac.uk). You won’t be able to respond to the texts, but you can contact Melanie at any stage. Please email Melanie if you wish to stop the texts.

Please see next page for the details of the experts who you will receive texts from.

Thank you very much for being an important part of this research study 😊

The ADAPT trial study team


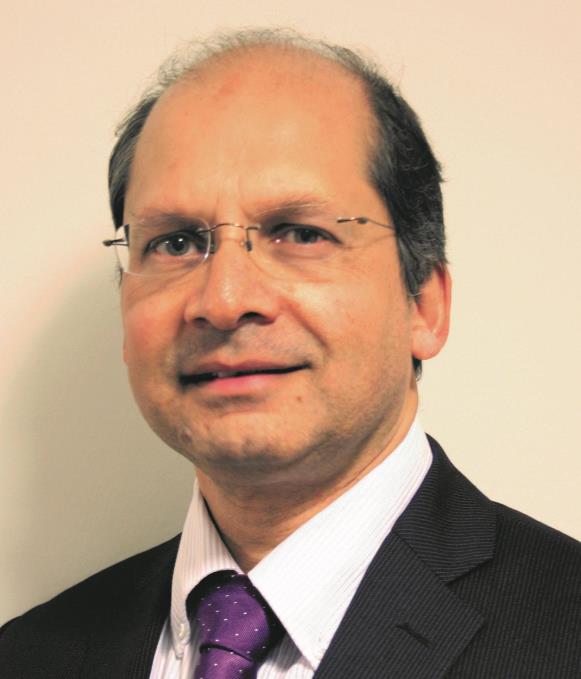
**People who you will receive texts and videos from**

Professor David D’Cruz is one of the top lupus specialists in the World. He is a rheumatology consultant at Guys and St Thomas’ hospital.

Prof D’C will be sending you texts with information and advice about lupus and managing symptoms.


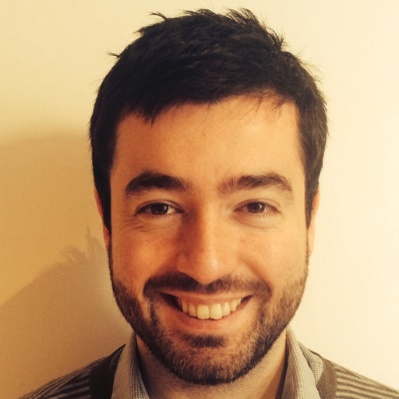


Dr Tom Pollak is a consultant neuropsychiatrist who works closely with patients with diseases affecting their brain and nervous system.

Dr Tom will be sending you texts with advice on mental health.


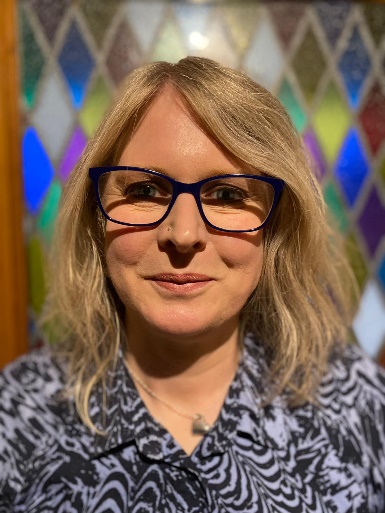


Wendy Diment has had lupus for over 10 years and has a lot of experience with living with the disease. She is also Vice Chair & Trustee for LUPUS UK, lupus Group Coordinator for Pembrokeshire & Carmarthenshire and Campaign Lead for the Fair Treatment for the Women of Wales

Wendy will be sending you texts with advice about quality of life, relationships and support.


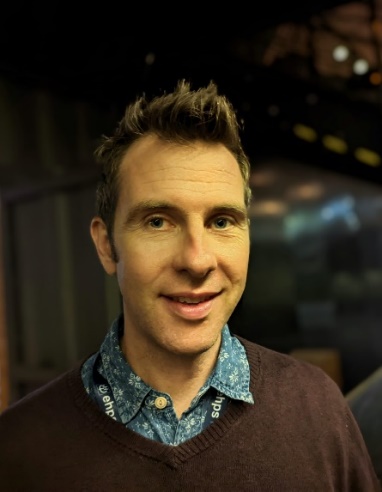


Felix Naughton is a health psychologist who is an expert on digital methods of improving health and behaviours to have better lives.

Felix will be sending you texts about positive changes you can make to help you adapt to the disease.

Please note: Texts have been written by these experts and with input from a large team of doctors and patients. They are automated and are not a substitute for direct medical advice from a doctor who knows you and your own specific disease symptoms. Please consult your own doctor for any medical advice.

**Fig A1 – Frequency of each intervention making participants feel better mentally**

**Fig A2- Frequency of each intervention making participants feel worse mentally**

**Fig A3 – Views on intervention duration**

**Table A1 -Regression analysis:changes in validated instruments after controlling for the following demographics: age, gender, time since diagnosis and ethnicity (Control group used as reference)**

| Validated Instrument | Text/video intervention | The Wren intervention | Pilates intervention |
| --- | --- | --- | --- |
| Depression : Baseline to F-up 1 | -2.25 | -2.67 | -3.14 |
| Depression: Baseline to F-up 2 | -2.17 | -2.21 | -1.81 |
| Resilience: Baseline to F-up 1 | 2.89 | 3.24 | 3.96 |
| Resilience: Baseline to F-up 2 | 1.81 | 2.69 | 4.16 |
| Loneliness: Baseline to F-up 1 | -1.34 | 0.15 | -0.11 |
| Loneliness: Baseline to F-up 2 | -0.11 | -0.39 | -0.61 |
| Qol: Baseline to F-up 1 | -0.46 | -0.62 | 0.51 |
| QoL: Baseline to F-up 2 | 0.35 | -1.50 | 0.53 |
| Fatigue: Baseline to F-up 1 | -1.40 | -3.04 | -4.85 |
| Fatigue: Baseline to F-up 2 | 1.20 | -1.67 | 0.09 |

**Fig A4 – The ADAPT survey instrument (items relevant to this study included)**

The ADAPT survey - About you  Please move the sliders to where best fits how you feel (please consider how you have felt over the past month on average)

|  | 0 | 10 | 20 | 30 | 40 | 50 | 60 | 70 | 80 | 90 | 100 |
| --- | --- | --- | --- | --- | --- | --- | --- | --- | --- | --- | --- |

| **1. Adapting.** How well do you feel you have adapted to the changes in your life from having a chronic disease: 0= Not adapted at all to 100= Fully adapted () | 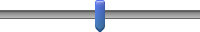 |
| --- | --- |
| **2. Coping.** How well do you feel you cope mentally with the challenges from your disease: 0= Not coping at all to 100 = Fully coping () | 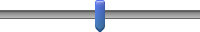 |
| **3. Control.** How in control of your life do you feel: 0= No control over my life to 100 = Full control () | 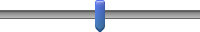 |
| **4. Knowledge**. How much knowledge do you feel you have about your disease: 0= No knowledge to 100 = Full knowledge of my disease () | 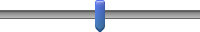 |
| **5. Confidence in self-managing**. How confident do you feel in self-managing your disease symptoms where appropriate: 0= No confidence to 100 = Fully confident to self-manage my symptoms where appropriate () | 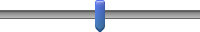 |
| **6. Self-esteem.** How do you feel your self-esteem is (confident in your own worth): 0=I feel completely worthless to 100=I am fully confident in my own worth () | 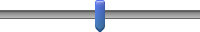 |
| **7. Satisfaction with life.** How do you feel OVERALL in terms of being satisfied with your life: 0= completely unsatisfied to 100 = Fully satisfied () | 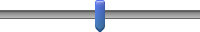 |

|  | 0 | 10 | 20 | 30 | 40 | 50 | 60 | 70 | 80 | 90 | 100 |
| --- | --- | --- | --- | --- | --- | --- | --- | --- | --- | --- | --- |

| **8. Participation in everyday life –** activities, socialising etc. How much do you feel you participate in everyday life: 0= No participation at all to 100= fully participate () | 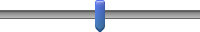 |
| --- | --- |
| **9. Community**. How much do you feel a part of a supportive community. 0=not a part of a community at all to 100 = fully a part of a supportive community () | 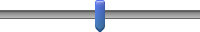 |

**Supplementary references:**

1. Sloan M, Wincup C, Harwood R, et al. Prevalence and identification of neuropsychiatric symptoms in systemic autoimmune rheumatic diseases: an international mixed methods study. *Rheumatology (Oxford)* 2023 20230726. DOI: 10.1093/rheumatology/kead369.

2. Wongpakaran N, Wongpakaran T, Pinyopornpanish M, et al. Development and validation of a 6-item Revised UCLA Loneliness Scale (RULS-6) using Rasch analysis. *Br J Health Psychol* 2020; 25: 233-256. 20200130. DOI: 10.1111/bjhp.12404.

3. Campbell-Sills L and Stein MB. Psychometric analysis and refinement of the Connor-davidson Resilience Scale (CD-RISC): Validation of a 10-item measure of resilience. *J Trauma Stress* 2007; 20: 1019-1028. DOI: 10.1002/jts.20271.

4. Kroenke K, Strine TW, Spitzer RL, et al. The PHQ-8 as a measure of current depression in the general population. *J Affect Disord* 2009; 114: 163-173. 20080827. DOI: 10.1016/j.jad.2008.06.026.

5. Machado MO, Kang NC, Tai F, et al. Measuring fatigue: a meta-review. *Int J Dermatol* 2021; 60: 1053-1069. 20201210. DOI: 10.1111/ijd.15341.

6. Feng YS, Kohlmann T, Janssen MF, et al. Psychometric properties of the EQ-5D-5L: a systematic review of the literature. *Qual Life Res* 2021; 30: 647-673. 20201207. DOI: 10.1007/s11136-020-02688-y.
